# Editorial: 15 years of frontiers in cellular neuroscience: blood brain barrier modulation and dysfunction in brain diseases

**DOI:** 10.3389/fncel.2024.1511314

**Published:** 2024-10-29

**Authors:** Duraisamy Kempuraj, Stefania Ceruti

**Affiliations:** ^1^Institute for Neuro-Immune Medicine, Dr. Kiran C. Patel College of Osteopathic Medicine, Nova Southeastern University, Ft. Lauderdale, FL, United States; ^2^Department of Pharmacological and Biomolecular Sciences “Rodolfo Paoletti”, Università degli Studi di Milano, Milan, Italy

**Keywords:** astrocytes, blood-brain barrier, endothelial cells, neurons, neurovascular unit, pericytes, tight junction proteins, neuroinflammatory disorders

## Introduction

The blood-brain barrier (BBB) is a dynamic structure that regulates transport (influx and efflux) of molecules between brain parenchyma and blood circulation and protects the brain from various pathogens, neurotoxic molecules from the periphery (Zapata-Acevedo et al., 2024) and drugs (Ronaldson and Davis, 2024). These protective actions are achieved due to the presence of tight junctions (TJ) with claudin 5, occluden, junctional adhesion molecules (JAMs), and zonula occludens-1 (ZO-1), adherens junctions (AJ) with Ve-Cadherin, catenin, and gap junctions (GJ) with connexins between adjacent endothelial cells that provide a physical barrier between brain parenchyma and peripheral blood (Kempuraj et al., 2020; Andjelkovic et al., 2023; Haruwaka et al., 2019). Microvascular endothelial cells in the brain express many transporters that regulate the transport of molecules, including glucose. Neurovascular unit (NVU) consists of microvascular endothelial cells, astrocytes, pericytes (overall forming the BBB), and neurons. An intact BBB maintains homeostasis of the brain microenvironment for normal neuronal signaling, while disrupted BBB, derangement of TJ proteins associated with increased BBB permeability are observed in many neuroinflammatory and neurodegenerative disorders (Sweeney et al., 2018; Kempuraj et al., 2020). Neuroinflammation plays an important role in various brain disorders such as chronic neurodegenerative diseases including Alzheimer's disease, Parkinson's disease, Multiple Sclerosis (MS), stroke, and traumatic brain injury (TBI) (Zapata-Acevedo et al., 2024). Systemic inflammatory disorders can cause and exacerbate neuroinflammatory responses in the brain (Paouri and Georgopoulos, 2019; Cheng et al., 2022). This Research Topic “*Blood-brain barrier modulation and dysfunction in brain diseases*” presents a collection of the following articles that demonstrate BBB disruption in the pathogenesis of brain disorders and how it can be therapeutically overcome.

## Significance and articles in this Research Topic

Gemfibrozil is a lipid-lowering drug that showed neuroprotective effects by inhibiting glial cells, inflammation, oxidative stress and apoptosis in neurodegenerative diseases (Ivraghi et al., 2024). A previous study has shown gemfibrozil's therapeutic beneficial effects in experimental autoimmune encephalomyelitis (EAE), an animal model of demyelinating disease MS (Dasgupta et al., 2007). Gemfibrozil is known to suppress the expression of various inflammatory molecules in glial cells through its binding to peroxisome proliferator-activated receptor alpha (PPARα). The paper by Mondal et al. for this Research Topic shows that gemfibrozil protects the integrity of the BBB/blood-spinal cord barrier (BSB), enhances Tregs, and decreases EAE pathogenesis through PPARβ, but not PPARα in EAE mice. Further, the above study indicates an important immunomodulatory role of gemfibrozil and PPARβ that may be explored for therapeutic intervention in MS (Mondal et al.).

Pericytes are important for BBB integrity and microvascular blood flow in the brain and thus contribute to the pathophysiology of NVU (Bhowmick et al., 2019). Pericytes are highly plastic and contractile cells that regulate blood flow in the brain microvessels, contribute to the maintenance of BBB integrity, angiogenesis, and control leukocyte transmigration (Alarcon-Martinez et al., 2021). In their review article for this Research Topic, Fu et al. highlight pericyte biomarkers, their stem cell role and contractile and paracrine functions, and their role in neuroinflammation especially following ischemic stroke. Further, these authors suggest that brain pericytes could be therapeutic targets for many brain disorders such as neurodegenerative disorders, stroke, and TBI (Fu et al.). This review article provides significant and current information on the pathophysiology of pericytes that are fundamental but still partially unknown contributors to NVU/BBB functions (Fu et al.).

Oxidative stress causes neuronal damage in brain disorders including TBI. Inhibition of histone deacetylases (HDACs) has been shown to decrease the level of oxidative stress by reducing the expression of reactive oxygen species (Lu et al., 2023). In the Research Topic paper, Inoue et al. studied the mechanism of how HDAC inhibition enhances BBB integrity using brain vascular endothelial cells. They report that incubation of immortalized human endothelial cells (HCMEC/D3) treated with W2A-16 (an HDAC inhibitor) retained the barrier property of endothelial cells as shown by increased trans-endothelial electrical resistance (TEER) and increased levels of BBB proteins as compared with untreated control cells. In this study, endothelial cells were cultured with hydrogen peroxidase and then the medium was switched to medium with or without W2A-16 and cultured. The above study concludes that HDAC inhibition by W2A-16 plays an important role in the formation of the BBB (Inoue et al.).

Microglia are brain-resident immune cells that present M0 (resting), M1 (proinflammatory), and M2 (anti-inflammatory) phenotypes (Wendimu and Hooks, 2022). Microglial activation is implicated in neuroinflammation and neurodegenerative diseases. A recent article discussed microglia and BBB in health and diseases (Mayer and Fischer, 2024). In a review article for this Research Topic, Deng et al. report crosstalk communication between the brain, heart, and spleen after stroke. These authors discuss how microglia-associated neuronal network inflammation induces cardiovascular disorders. Further, they indicate that these effects involve BBB disruption associated with increased permeability, entry of peripheral immune cells into the brain and entry of brain-derived molecules into the peripheral circulation and peripheral organs and activation of monocytes (Deng et al.).

Detection of neuroinflammatory biomarkers in cerebrospinal fluid (CSF), blood, and brain tissues, and their validation in *in vitro* cell culture disease models are useful for diagnoses and for assessing the severity of neuroinflammatory and neurodegenerative disorders especially associated with NVU/BBB components (Kadry et al., 2020). The review article by Kempuraj et al. on this Research Topic provides an overview of the most important markers for NVU/BBB, neuroinflammatory and neurodegenerative disorders expressed by various brain cells (Kempuraj et al.). These authors indicate that derangements and damage to the TJ, AJ, and GJ components of the BBB lead to increased BBB permeability, resulting in edema, increased neuroinflammation and neuronal damage in various brain disorders (Kempuraj et al.). Additionally, this review article highlights that BBB-on-a-chip modeling offers promising potential for the pre-clinical in-depth understanding of NVU/BBB brain pathologies and neurotherapeutics.

## Concluding remarks

Neuroinflammatory and neurodegenerative disorders show NVU/BBB dysfunctions and increased BBB permeability. Understanding the mechanisms underlying these pathological events is important for the assessment of disease severity and to identify innovative treatment options for brain disorders. This Research Topic provides several insights on the NVU/BBB which could lead to the development of more effective therapeutic options for these disorders.
